# A scalable method for identifying frequent subtrees in sets of large phylogenetic trees

**DOI:** 10.1186/1471-2105-13-256

**Published:** 2012-10-03

**Authors:** Avinash Ramu, Tamer Kahveci, J Gordon Burleigh

**Affiliations:** 1 Electrical and Computer Engineering, University of Florida, Gainesville, FL, USA; 2 Computer and Information Science and Engineering, University of Florida, Gainesville, FL, USA; 3 Department of Biology, University of Florida, Gainesville, FL, USA

**Keywords:** Phylogenetic trees, Frequent subtree

## Abstract

**Background:**

We consider the problem of finding the maximum frequent agreement subtrees (MFASTs) in a collection of phylogenetic trees. Existing methods for this problem often do not scale beyond datasets with around 100 taxa. Our goal is to address this problem for datasets with over a thousand taxa and hundreds of trees.

**Results:**

We develop a heuristic solution that aims to find MFASTs in sets of many, large phylogenetic trees. Our method works in multiple phases. In the first phase, it identifies small candidate subtrees from the set of input trees which serve as the seeds of larger subtrees. In the second phase, it combines these small seeds to build larger candidate MFASTs. In the final phase, it performs a post-processing step that ensures that we find a frequent agreement subtree that is not contained in a larger frequent agreement subtree. We demonstrate that this heuristic can easily handle data sets with 1000 taxa, greatly extending the estimation of MFASTs beyond current methods.

**Conclusions:**

Although this heuristic does not guarantee to find all MFASTs or the largest MFAST, it found the MFAST in all of our synthetic datasets where we could verify the correctness of the result. It also performed well on large empirical data sets. Its performance is robust to the number and size of the input trees. Overall, this method provides a simple and fast way to identify strongly supported subtrees within large phylogenetic hypotheses.

## Background

**Figure 1:**
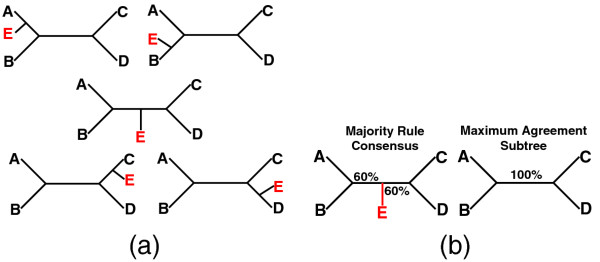
**(a) A collection of five input trees.** The same subtree with taxa A, B, C, and D is present in all input trees, and only the position of taxa E changes. **(b)** The majority rule consensus and maximum agreement subtrees of the 5 input trees in Figure 1a.

Phylogenetic trees represent the evolutionary relationships of organisms. While recent advances in genomic sequencing technology and computational methods have enabled construction of extremely large phylogenetic trees (e.g., [1-3]), assessing the support for phylogenetic hypotheses, and ultimately identifying well-supported relationships, remains a major challenge in phylogenetics. Support for a tree often is determined by methods such as nonparametric bootstrapping [4], jackknifing [5], or Bayesian MCMC sampling (e.g., [6]), which generate a collection of trees with identical taxa representing the range of possible phylogenetic relationships. These trees can be summarized in a consensus tree (see [7]). Consensus methods can highlight support for specific nodes in a tree, but they also may obscure highly supported subtrees. For example, in Figure 1, the subtree containing taxa A, B, C, and D is present in all five input trees. However, due to the uncertain placement of taxon E, the majority rule consensus tree implies that the clades in the tree have relatively low (60%) support.

Alternate approaches have been proposed to reveal highly supported subtrees. The maximum agreement subtree (MAST) problem seeks the largest subtree that is present in all members of a given collection of trees [8]. For example, in Figure 1 the MAST includes taxa A, B, C, and D. Finding the MAST is an NP-hard problem [9], although efficient algorithms exist to compute the MAST in some cases (e.g., [9-17]). In practice, since any difference in any single tree will reduce the size of the MAST, the MAST is often quite small, limiting it usefulness.

A less restrictive problem is to find frequent agreement subtrees (FAST), or subtrees that are found in many, but not necessarily all, of the input trees (see [18]). In this problem, a subtree is declared as frequent if it is in at least as many trees as a user supplied frequency threshold. Several algorithmic approaches have been suggested to identify FASTs, and specifically the maximum FASTs (MFASTs), or FASTs that contain the largest number of taxa. A variant of this problem seeks the maximal FASTs, i.e., FASTS that are not contained in any other FASTs. Notice that an MFAST is a *maximal* FAST, however, the inverse is not necessarily true. Zhang and Wang defined algorithms, implemented in Phylominer, to identify FASTs from a collection of phylogenetic trees [19,20]. These algorithms are guaranteed to find all FASTs but they may be prohibitively slow for data sets larger than 20 taxa. Cranston and Rannala implemented Metropolis-Hastings and Threshold Accepting searches to identify large FASTs from a Bayesian posterior distribution of phylogenetic trees [21]. This approach can handle thousands of input trees but it may not be feasible if the trees have more than 100 taxa [21].

Another approach to reveal highly supported subtrees from a collection of trees is to identify and remove rogue taxa, or taxa whose position in the input trees is least consistent. Recently, several methods have been developed that can identify and remove rogue taxa from collections of trees with thousands of taxa [22-24]. However, unlike MAST or FAST approaches, they do not provide guarantees about the support for the remaining taxa.

In this paper, we describe a heuristic approach for identifying MFASTs in collections of trees. Unlike previous methods, our method easily scales to datasets with over a thousand taxa and hundreds of trees. Towards this goal, we develop a heuristic solution that works in multiple phases. In the first phase, it identifies small candidate subtrees from the set of input trees which serve as the seeds of larger subtrees. In the second phase, it combines these seeds to build larger candidate MFASTs. In the final phase, it performs a post processing step. This step ensures that the size (i.e., number of taxa) of the FAST found can not be increased further by adding a new taxon without reducing its frequency below a user supplied frequency threshold. We demonstrate that this heuristic can easily handle data sets with 1000 taxa. We test the effectiveness of these approaches on simulated data sets and then demonstrate its performance on large, empirical data sets. Although our heuristic does not guarantee to find all MFASTs or the largest MFAST in theory, it found the true MFAST in all of our synthetic datasets where we could verify the correctness of the result. It also performed well on the empirical data sets. Its performance is robust with respect to the number of input trees and the size of the input trees.

## Methods

In this section we describe our method that aims to find *Maximum Frequent Agreement SubTrees (MFASTs)* in a given set of *m* phylogenetic trees T= {*T*_1_, *T*_2_, …, *T*_*m*_}. Our method follows from the observation that an MFAST is present in a large number of trees in T. The method builds MFASTs bottom up from small subtrees of taxa in the trees in T. Briefly, it works in three phases. 

• **Phase 1.** Seed generation (Section “Phase one: Seed generation”).In the first phase, we identify small subtrees from the input trees that have a potential to be a part of an MFAST. We call each such subtree a *seed*.

• **Phase 2.** Seed combination (Section “Phase two: Seed combination”).In the second phase, we construct an initial FAST by combining the seeds found in the first phase.

• **Phase 3.** Post processing (Section “Phase three: Post-processing”).In the third phase, we grow the FAST further to obtain the maximal FAST that contains it by individually considering the taxa which are not already in the FAST. We report the resulting maximal FAST as a possible MFAST.

First, we present the the basic definitions needed for this paper in Section “Preliminaries and notation”. We then discuss each of the three phases above in detail.

### Preliminaries and notation

**Figure 2:**
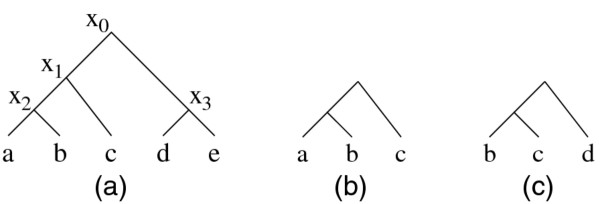
**(a) A rooted, bifurcating phylogenetic tree *T *built on five taxa labeled with *a, b, c, d *and *e***. The internal nodes are shown with *x*_0_, *x*_1_, *x*_2_ and *x*_3_. **(b)** A clade of *T* rooted at *x*_1_. **(b)** and **(c)** Two subtrees of *T* by contracting the taxa sets {*d, e*} and {*a, e*}.

In this section, we present the key definitions and notations needed to understand the rest of the paper. We describe our method using rooted and bifurcating phylogenetic trees. However, our method and definitions can easily be applied to unrooted or multifurcating trees with minor or no modifications. Also, we assume that all the taxa are placed at the leaf level nodes of the phylogenetic tree, and all the internal nodes are inferred ancestors. Figure 2(a) shows a sample phylogenetic tree built on five taxa. We define the *size* of a tree as the number of taxa in that tree. We start by defining key terms.

#### Definition 1 (**Clade**)

Let *T* be a phylogenetic tree. Given an internal node of *T*, we define the set of all nodes and edges of *T* contained under that node as the *clade* rooted at that node.

Each internal node of a phylogenetic tree corresponds to a clade of that tree. Figure 2(b) depicts the clade of the tree in Figure 2(a) rooted at *x*_1_.

#### Definition 2 (**Contraction**)

Let *T* be a phylogenetic tree with *n* taxa. The contraction operation transforms *T* into a tree with *n*−1 taxa by removing a given taxon in *T* along with the edge that connects that taxon to *T*.

The contraction operation can extract the clades of a tree by removing all the taxa that are not a part of that clade. It can also extract parts of the tree that are not necessarily clades. We use the term *subtree* to denote a tree that is obtained by applying contractions to arbitrary set of taxa in a given tree. Formal definition is as follows.

#### Definition 3 (**Subtree**)

Let *T* and *T’* be two phylogenetic trees. We say that *T’* is a *subtree* of *T* if *T* can be transformed into *T’* by applying a series of contractions on *T*.

If a tree *T’* is a subtree of another tree *T*, we say that *T’* is *present* in *T*. Notice that a clade is always a subtree, but the inverse is not true all the time. Figures 2(b) and 2(c) illustrate two subtrees of the tree in Figure 2(a). Let us denote the number of combinations of *k* taxa from a set of *n* taxa with nk. In general, if a tree has *n* taxa, then that tree contains nk subtrees with *k* taxa. As a consequence, that tree contains 2^*n*^ − 1 subtrees of any size including itself.

#### Definition 4 (**Frequency**)

Let T = {*T*_1_, _*T*2_, … , *T*_*m*_} be a set of *m* phylogenetic trees and *T* be a phylogenetic tree. Let us denote the number of trees in T at which *T* is present with the variable *m’*. We define the frequency of *T* in T as

$$\;\text{freq}\left(T,T\right)=\frac{m^{\prime }}{m}.$$

#### Definition 5 (**FAST**)

Let T = {*T*_1_, *T*_2_, … , *T*_*m*_} be a set of *m* phylogenetic trees and *T* be a phylogenetic tree. Let *γ* be a number in [0, 1] interval that denotes frequency cutoff. We say that *T* is a Frequent Agreement SubTree (FAST) of T if its frequency in T is at least *γ* (i.e., freq(T,T)≥γ).

We say that a FAST is *maximal* if there is no other FAST that contains all the taxa in that FAST. Clearly, larger FASTs indicate biologically more relevant consensus patterns. The following definition summarizes this.

#### Definition 6 (**MFAST**)

Let T = {*T*_1_, *T*_2_, …, *T*_*m*_} be a set of *m* phylogenetic trees. Let *γ* be a number in [0, 1] interval that denotes frequency cutoff. A FAST *T* of T is a Maximum Frequent Agreement SubTree (MFAST) of T if there is no other FAST *T’* of T that has a larger size than *T*.

Formally, given a set of phylogenetic trees T = {*T*_1_, *T*_2_, …, *T*_*m*_} and a frequency cutoff, *γ*, we would like to find the MFASTs in T in this paper. We develop an algorithm that aims to solve this problem. Table 1 lists the variables used throughout the rest of this paper.

**Table 1:** Commonly used variables and functions in this paper

	
T	A set of phylogenetic trees
*T*_*i*_	*i*th tree
*m*	Number of trees in T
*n*	Number of taxa in each input tree
*a*_*i*_	*i*th taxa
freq(T,T)	Frequency of the subtree *T* in T
*γ*	Frequency cutoff
*S*_*i*_	*i*th seed (each seed is a subtree of a tree in T)
*k*	Size of a seed
*c*	Number of contractions used to create a seed

### Phase one: Seed generation

The first phase extracts small subtrees from the given set of trees. From these subtrees we extract the basic building blocks which are used to construct MFASTs. We call these building blocks seeds. Conceptually each seed is a phylogenetic tree that contains a small subset of the taxa that make up the trees in T. We characterize each seed with three features that are listed below. We elaborate on each feature later in this section. 

1. *Seed size (k)* is the number of taxa in the seed.

2. Number of contractions (*c*) is the number of taxa we prune from a clade taken from an input tree in order to extract the seed.

3. *Frequency (f)* is the fraction of input trees in which the seed is present.

**Figure 3:**
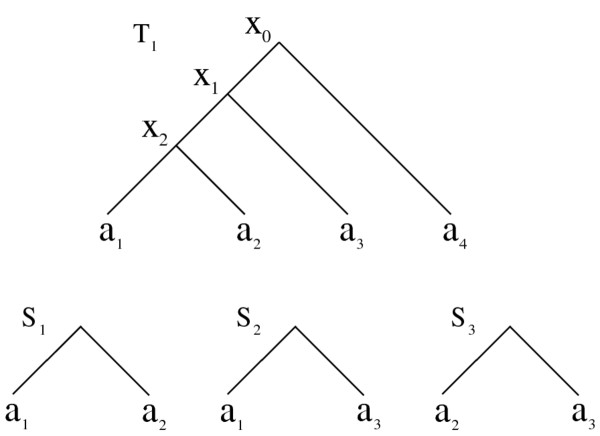
***T***_**1**_** is an input tree built on four taxa *a***_**1**_, ***a***_**2**_, ***a***_**3**_**and *a***_**4**_. The internal nodes of *T*_1_ are labeled as *x*_0_, *x*_1_ and *x*_2_. *S*_1_ is the only seed obtained from *T*_1_ when *k* = 2 and *c* = 0. That is *S*_1_ is identical to the clade rooted at *x*_2_. *S*_1_, *S*_2_ and *S*_3_ are the seeds extracted from *T*_1_ when *k* = 2 and *c* = 1. They are all extracted from the clade rooted at *x*_1_ by contracting *a*_3_, *a*_2_ and *a*_1_ respectively.

**Figure 4:**
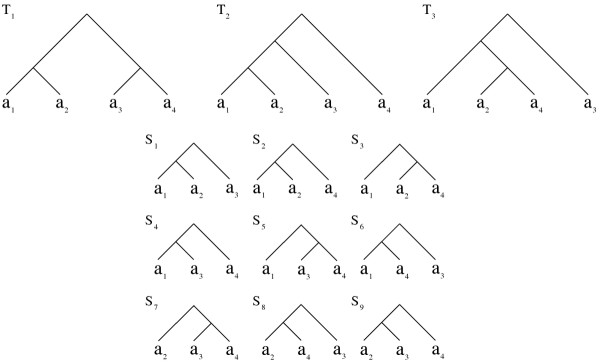
**The set of input trees *T***_**1**_, ***T***_**2**_, ***T***_**3**_** and the set of all nine potential seeds *S***_**1**_, ***S***_**2 **_***… S***_**9**_** when the seed characteristics are set to *k* = 3 and *c = 1***. All the potential seeds have three taxa as k = 3. We need one contraction from the input tree to obtain each seed. *S*_1_ has frequency 1.0 as it is present in *T*_1_, *T*_2_ and *T*_3_. Seed *S*_2_ has frequency ∼0.67 as it is present in *T*_1_ and *T*_2_. Remaining seeds have frequency ∼0.33 as each appears in only one of the three trees.

We explain the seed features with the help of Figures 3 and 4. The first two characteristics explain how a seed can be found in one of the trees in T. They indicate that there is a clade of a tree in T such that this clade contains *k* + *c* taxa and it can be transformed into that seed after *c* contractions from that clade. For instance in Figure 3, when *k* = 2 and *c* = 0, only seed *S*_1_ can be extracted from *T*_1_ by choosing the clade rooted at *x*_2_. When *k* = 2 and *c* = 1, seeds *S*_1_, *S*_2_ and *S*_3_ can be obtained using one contraction (*a*_3_, *a*_2_ and *a*_1_ respectively) from the clade rooted at *x*_1_.

The last feature denotes the number of trees in T in which the seed is present. For example in Figure 4, there are nine seeds *S*_1_, *S*_2_, …, *S*_9_ extracted from the three input trees using only one contraction. Among these, the frequency of *S*_1_ is 1 as it is present in all the trees. Frequency of *S*_2_ is about 0.67 for it is present in only two out of three trees (*T*_1_ and *T*_2_). The frequency of the rest of the seeds is only about 0.33. Recall that, by definition, an MFAST is present in at least a fraction *γ* of the trees in T. Therefore, we consider only the seeds whose frequency values are equal to or greater than this number ( i.e. , *f* ≥ *γ*).

Given the values of *k*, *c* and *γ*, we extract all the seeds which possess the desired feature values from the set of input trees as follows. In the newick string representation of a tree, a pair of matching parentheses corresponds to an internal node in the tree. The number of taxa in the clade rooted at this internal node is given by the number of labels between the two matching parentheses. Following from this observation, we scan the newick string of each tree one by one. For each such tree, we identify the clades which have *k* + *c* taxa. Notice that, if a tree contains *n* taxa, then it contains at most $\frac{n}{k+c}$ clades of size *k* + *c* as no two such clades can contain common taxa. We then extract all combinations of *k* taxa from each of these clades by contracting the remaining *c* taxa. The number of ways this can be done is k+cc. Notice that all the small trees extracted this way possess the first two characteristics explained above. At this point, we however do not know their frequencies. Therefore, we call them *potential seeds*. It is worth mentioning that the same seed might be extracted from different trees. As we extract a new potential seed, before storing it in the list of potential seeds, we check if it is already present there. We include it in the potential seed list only if it does not exist there yet. Otherwise, we ignore it. This way, we maintain only one copy of each seed.

Once we build our potential seed list for all the trees in T, we go over them one by one and count their frequency in T as the fraction of trees that contain them. We filter all the potential seeds whose frequencies are less than the frequency cutoff. We keep the remaining ones as the list of seeds along with the frequency of each seed.

In Figure 4, consider the tree *T*_1_ that has four taxa. For *k* = 3 and *c* = 1, there is only one clade of size *k* + *c* = 4 which is the tree *T*_1_ itself. We extract four potential seeds, each having three leaves from this tree. The potential seeds in this figure are given by *S*_1_, *S*_2_, *S*_5_ and *S*_7_ which we extract by contracting *a*_4_, *a*_3_, *a*_2_ and *a*_1_ respectively from *T*_1_.

### Phase two: Seed combination

At the end of the first phase, we obtain a set of frequent seeds from the input trees. Notice that each seed is a FAST as each seed is present in sufficient number of trees specified by *γ*. These seeds are the basic building blocks of our method. In the second phase of our method, we combine subsets of these seeds to construct larger FASTs.

We first define what it means to combine two seeds. In order to combine two seeds, it is a necessary condition that both seeds are present in at least one common tree *T* in T. We call such a tree *T* as the *reference tree*. We combine two seeds with the guidance of a reference tree. Let *S*_1_ and *S*_2_ be two seeds and let *T* be their reference tree. Let *L*_1_, *L*_2_ and *L* be the set of taxa in *S*_1_, *S*_2_ and *T* respectively. Combining *S*_1_ and *S*_2_ results in the tree that is equivalent to the one obtained by contracting the taxa in *L* − (*L*_1_ ∪ *L*_2_) from *T*. For simplicity, we will denote the combine operation using *T* as the reference network with the ⊕_*T*_ symbol. For instance we denote combining *S*_1_ and *S*_2_ with *T* being the reference tree as *S*_1_ ⊕ _*T*_*S*_2_. To simplify our notation, whenever the identity of the reference tree is irrelevant, we will use the symbol ⊕ instead of ⊕_*T*_.

**Figure 5:**
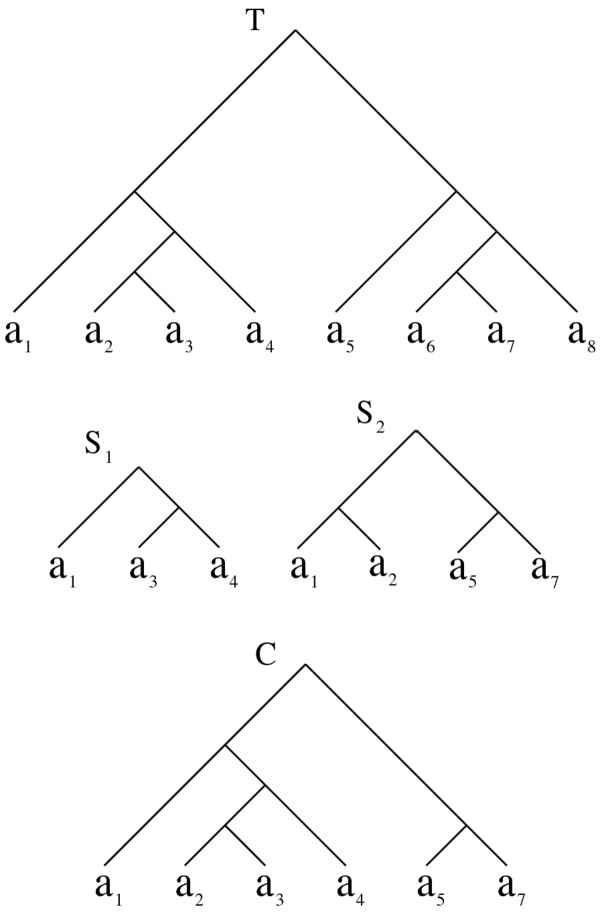
***T*is the reference tree. ***S*_1_ and *S*_2_ are the seeds to be combined, both are present in *T*. *C* is obtained by pruning the subtree containing taxa *a*_1_, *a*_2_, *a*_3_, *a*_4_, *a*_5_ and *a*_7_ from *T*.

Figure 5 demonstrates how two seeds *S*_1_ and *S*_2_ are combined with the help of the reference tree *T*. In this figure, both *S*_1_ and *S*_2_ are subtrees of *T*. Thus, it is possible to use *T* as the reference tree. We have *L*_1_ = {*a*_1_,*a*_3_,*a*_4_}, *L*_2_ = {*a*_1_,*a*_2_,*a*_5_,*a*_7_}. Thus, we build *C* = *S*_1_ ⊕ _*T*_*S*_2_ by contracting the taxa in *L* − (*L*_1_ ∪ *L*_2_) = {*a*_6_,*a*_8_} from *T*.

So far, we have explained how to combine two seeds *S*_1_ and *S*_2_ using a reference tree. It is possible that many trees in T have both seeds present in them. Thus, one question is which of these trees should we use as the reference tree to combine the two seeds? The brief answer is that all such trees need to be considered. However, we make several observations that helps us avoid combining *S*_1_ and *S*_2_ using each such reference tree one by one exhaustively without ignoring any of such trees. We explain them next.

Consider two trees *T*_1_ and *T*_2_ from T where both seeds are present in. There are two cases for *T*_1_ and *T*_2_. 

• Case 1: *S*_1_ ⊕_*T*_1__*S*_2_ = *S*_1_ ⊕_*T*_2__*S*_2_. In this case, it does not matter whether we use *T*_1_ or *T*_2_ as the reference tree. They will both lead to the same combined subtree. Thus, we use only one.

• Case 2: *S*_1_ ⊕_*T*_1__*S*_2_ ≠ *S*_1_ ⊕_*T*_2__*S*_2_. In this case, the trees *T*_1_ and *T*_2_ lead to alternative combination topologies. So, we consider both of them separately.

We utilize the observations above as follows. We start by picking one reference tree arbitrarily. Once we create a combined subtree using that tree, we check whether that subtree is present in the remaining trees in T. We mark those trees that contain it as considered for reference tree and never use them as reference for the same seed pair again. This is because those trees fall into the first case described above. This way, we also store the frequency of the combined subtree in T. If the number of unmarked trees is too small (i.e., less than *γ* × *m*) then it means that even if all the remaining trees agree on the same combined topology for the two seeds under consideration, they are not sufficient to make it a FAST. Thus, we do not use any of the remaining trees as reference for those two seeds. Otherwise, we pick another unmarked tree arbitrarily and repeat the same process until we run out of reference trees.

The next question we need to answer is which seed pairs should we combine? To answer this question we first make the following proposition.

#### Proposition 1

Assume that we are given a set of phylogenetic trees T. Let *S*_1_ and *S*_2_ be two seeds constructed from the trees in T. For all trees T∈T, we have the following inequality

$$\;\text{freq}(S_{1}{⊕}_{T}S_{2},T)\leq \text{min}\{\text{freq}(S_{1},T),\;\text{freq}(S_{2},T)\}$$

#### Proof

For any *T*, both *S*_1_ and *S*_2_ are subtrees of *S*_1_ ⊕_*T*_*S*_2_. Thus if *S*_1_ ⊕_*T*_*S*_2_ is present in a tree, then both *S*_1_ and *S*_2_ are present in that tree. As a result, *freq*(*S*_1_ ⊕_*T*_*S*_2_, T) ≤ *freq*(*S*_1_, T) and *freq*(*S*_1_ ⊕_*T*_*S*_2_, T) ≤ *freq*(*S*_2_, T). Hence,

$$\;\text{freq}(S_{1}{⊕}_{T}S_{2},T)\leq \text{min}\{\text{freq}(S_{1},T),\;\text{freq}(S_{2},T)\}$$

□

Proposition 1 states that as we combine pairs of seeds to grow them, their frequency monotonically decreases. This suggests that it is desirable to combine two seeds if both of them have large frequencies. This is because if one of them has a small frequency, regardless of the frequency of the other, the combined tree will have a small frequency. As a result its chance to grow into a larger tree through additional combine operations gets smaller. Following this intuition, we develop two approaches for combining the seeds. 

1. *In-order Combination* (Section “In-order combination”).

2. *Minimum Overlap Combination* (Section “Minimum overlap combination”).

Both approaches accept the list of seeds computed in the first phase as input and produce a larger FAST that is a combination of multiple seeds. Both of them also assume that the list of input seeds are already sorted in decreasing order of their frequencies. We discuss these approaches next.

#### In-order combination

The in-order combination approach follows from Proposition 1. It assumes that the seeds with higher frequencies have greater potential to be a part of an *MFAST*. It exploits this assumption as follows, first it picks a seed as the starting point to create a FAST. It then grows this seed by combining it with other seeds starting from the most frequent one as long as the frequency of the resulting tree remains at least as large as the given cutoff *γ*. It repeats this process by trying each seed as the starting point, Algorithm Algorithm 1 In order combination presents this approach.

### Algorithm 1 In order combination

*FAST* ← *∅*

**for all** seeds *S*_*i*_**do**

*FAST*^*′*^ ← *S*_*i*_

Mark *S*_*i*_ as considered

repeat

*S*_*j*_ ← seed with highest frequency among unconsidered seeds Mark *S*_*j*_ as considered CUTOFF ← *γt*_*FAST*^*′*^ ← *FAST*^*′*^

repeat

Pick the next unconsidered tree T∈T as reference

Mark all the trees as that contain *FAST*^*′*^ ⊕_*T*_*S*_*j*_ as considered

**if** freq($\text{FAS}T^{\prime }{⊕}_{T}S_{j},T)\geq$ CUTOFF **then**

*t*_*FAST*^*′*^ ← *FAST*^*′*^ ⊕_*T*_*S*_*j*_

CUTOFF ← freq($\text{FAS}T^{\prime }{⊕}_{T}S_{j},T)$

end if

**until** Less than *γ* × *m* unmarked reference trees are left in T

*FAST*^*′*^←*t*_*FAST*^*′*^

Unmark all trees in T

**until** all seeds are considered

**if** size of *FAST*^*′*^ ≥ size of *FAST***then**

*FAST* ← *FAST*^*′*^

end if

Unmark all seeds

end for

In Algorithm Algorithm 1 In order combination we first initialize the FAST as empty. We then consider each seed one by one. We initialize a temporary subtree denoted by *FAST’* with the seed *S_*i*_* under consideration and mark *S*_*i*_ as considered. We combine the FAST’ with a seed *S*_*j*_ which has the highest frequency amongst the seeds that have not been added. If multiple seeds have the highest frequency, we randomly pick one of them and mark that seed *S_*j*_* as added to the FAST’. There can be alternative ways to combine FAST’ with *S*_*j*_ leading to different topologies. We use the trees in T that contain both FAST’ and *S*_*j*_ as guides to try only the topologies that exist in T. We stop constructing alternative topologies as soon as we ensure that there are not sufficient number of trees to yield frequency of *γ*. We set FAST’ to the combined seed if the combined seed has large enough frequency. We then consider the seed with the next highest frequency for addition and repeat this step till all *S_*j*_* have been considered. If the resulting temporary FAST is larger than FAST we replace the smaller FAST with the larger one. In the next iteration, we initialize the *FAST* with the next *S_*i*_*. Using this approach we can initialize the *FAST* with all *S_*i*_*, alternatively if the user wishes to limit the amount of time spent using a *maximum time cutoff * we stop the outermost loop (i.e., alternative initializations of FAST’) as soon as the allowed running time budget is reached.

Notice that in Algorithm 3 each seed *S*_*i*_ can lead to a different FAST. We record only the FAST that has the largest size. However, it is trivial to maintain the top *k* FASTs with the largest size instead if the user is looking for *k* alternative maximal FASTs.

#### Minimum overlap combination

The purpose of combining seeds is to construct a FAST that is large in size. Our in-order combination approach (Section “In-order combination”) aimed to maximize the frequency of the combined seeds. In this section, we develop our second approach, named *Minimum Overlap Combination*. This approach picks seeds so that their combination produced as large subtree as possible. We elaborate on this approach next.

When we combine two seeds, the size of the resulting tree becomes at least as big as the size of each of these seeds. Formally let *S*_1_ and *S*_2_ be two seeds (i.e., trees). Let *L*_1_ and *L*_2_ be the set of taxa combined in *S*_1_ and *S*_2_. We denote the size of a set, say *L*_1_, with |*L*_1_|. The size of the tree resulting from combination of *S*_1_ and *S*_2_ is |*L*_1_| + |*L*_2_| − |*L*_1_ ∩ *L*_2_|. For a given fixed seed size, the first two terms of this formulation remains unchanged regardless of the seed. The last term determines the growth in the size of the FAST. Thus, in order to grow the FAST rapidly, it is desirable to combine two frequent subtrees with a small number of common taxa.

Our second approach follows from the observation above. We introduce a criteria called the *overlap* between two subtrees as the number of taxa common between them. Our minimum overlap combination approach works the same as Algorithm Algorithm 1 In order combination with a minor difference in selecting the seed *S*_*j*_ that will be combined with the current temporary FAST (i.e., FAST’). Rather than choosing the seed with the largest frequency, this approach chooses the one that has the least overlap with FAST’ among all the unconsidered and frequent seeds. If multiple seeds have the same smallest overlap, it considers the frequency as the tie breaker and chooses the one with the largest frequency among those.

### Phase three: Post-processing

So far we described how to obtain seeds (Section “Phase one: Seed generation”) and how to combine them to construct FAST (Section “Phase two: Seed combination”). The two approaches we developed for combining seeds aim to maximize the size of FAST. However, they do not ensure the maximality of the resulting FAST. There are two main reasons that prevent our seed combining algorithms from constructing maximal FAST. First, some of the taxa of a maximal FAST may not appear in any seed (i.e. false negatives). As a result no combination of seeds will lead to that maximal FAST. Second, even if all the taxa of a maximal FAST are parts of at least one seed, our algorithms will reject combining that seed with the FAST of the seeds if those seeds contain other taxa that are not part of the maximal FAST (i.e. false positives).

In the post-processing phase, we tackle above-mentioned problem. Algorithm 3 describes the post processing phase in detail. We do this by considering all taxa which are not already present in the *FAST* one by one. We iteratively grow the current FAST by including one more taxon at a time if the frequency of the resulting FAST remains at least as large as the frequency cutoff *γ*. We repeat these iterations until no new taxon can be included in the FAST. Thus the resulting FAST is guaranteed to be maximal.

### Algorithm 2 Post processing

INPUT = FAST from the seed combination phase

INPUT = T

OUTPUT = Maximal FAST

RESULT ← FAST

**for all ***a*_*i*_ not in FAST **do**

CUTOFF ← *γ*

t_RESULT ← *RESULT*

repeat

Pick the next unconsidered tree T∈T as reference

RESULT’ ← RESULT ⊕_*T*_*a*_*i*_

Mark all the trees that contain RESULT’ as considered

**if** frequency of RESULT’ ≥ CUTOFF **then** t_RESULT ← RESULT’ CUTOFF ← frequency of RESULT’

end if

**until** Less than *γ* × *m* unmarked reference trees are left in T

RESULT ← t_RESULT

Unmark all trees in T

end for

**return** RESULT

We expect the post processing step to identify quickly the taxa that have a potential to be in an MFAST that might have not been considered during the seed generation and seed combination phases. At the end of the post processing step we obtain an MFAST.

### Complexity analysis of our method

In this section we discuss the complexity of our method in terms of the three phases involved in it. Let T be a set of *m* phylogenetic trees having *n* leaves each. The complexity of the different phases of our method are as follows.

#### Phase one

Finding the seeds involves enumerating all the subtrees and checking their frequencies. Given seed size *k* and number of contractions *c*, each tree will contain at most $\frac{n}{k+c}$ clades each leading to k+cc alternative subtrees. Thus, in total there can be up to mnk+ck+cc seeds (possibly many of them identical) from all the trees in T. Typically, the values of *k* and *c* are fixed and small (in our experiments we have *k* ∈ {3, 4, 5} and *c* ∈ {0, 1, 2, 3, 4, 5}) leading to *O*(*mn*) seeds.

The complexity of finding whether a seed is present in a single tree is *O*(*n* log *n*). Given that there are *m* trees in T, the cost of computing the frequency of a single seed is *O*(*mn* log *n*). Thus, the time complexity for finding the frequency of all the seeds is this expression multiplied by the number of seeds, which is *O*(*m*^2^*n*^2^ log *n*).

#### Phase two

Consider a set of *p* frequent seeds that will be considered for combining in this phase. Recall that we have two approaches to combine them. Below, we focus on each.

INORDER COMBINATION We try to combine each seed with every other seed leading to *O*(*p*^2^) iterations. The complexity of checking the frequency of each combined subtree is *O*(*mn*log*n*). Also, there can be up to *O*(*m*) different reference trees for guiding the combine operation. Multiplying these terms, we obtain the complexity of phase using this approach as *O*(*p*^2^*m*^2^*n* log *n*).

MINIMUM OVERLAP COMBINATION The complexity of combining the frequent seeds using the minimum overlap combination approach is very similar to the inorder approach except for an additional term. The additional complexity is because we maintain the overlap between the subtrees. This leads to the complexity *O*(*p*^2^*n*^2^ + *p*^2^*m*^2^*n* log *n*).

#### Phase three

Here, we consider the FAST obtained from each of the *p* frequent seeds in phase two. For each FAST, we sequentially go over each taxa one by one leading to *O*(*n*) iterations. There can be up to *O*(* γ * × *m*) references to add a taxon. So the cost of extending all *p* FASTs is *O*(*γ* × *mnp*).

Notice that each frequent seed has to appear in at least *γ* × *m* trees. Thus, the number of unique frequent seeds *p* is bounded by $O(\frac{\mathrm{mn}}{\gamma \times m})$= $O(\frac{n}{\gamma })$. Thus, adding the cost of all the three phases, the overall time complexity of our method using inorder combination is

$$\;O(m^{2}n^{2}\text{log}n+\frac{m^{2}n^{3}\text{log}n}{\gamma^{2}}+mn^{2}).$$

That using minimum overlap combination is

$$\;O(m^{2}n^{2}\text{log}n+\frac{m^{2}n^{3}\text{log}n}{\gamma^{2}}+\frac{n^{4}\text{log}n}{\gamma^{2}}+mn^{2}).$$

In the two summations above, the second term is asymptotically larger than the first and the last terms. Thus, we can simplify the asymptotic time complexity of inorder and minimum overlap combinations as

$$\;O(\frac{m^{2}n^{3}\text{log}n}{\gamma^{2}})$$

and

$$\;O(\frac{n^{3}\text{log}n}{\gamma^{2}}(m^{2}+n))$$

respectively.

## Results and discussion

This section evaluates the performance of our MFAST algorithm experimentally.

### Implementation details

We implemented our MFAST algorithm using C and Perl. More specifically, we implemented the first two phases (seed generation and seed combination) in C and the third phase (post processing) in Perl. We utilize the functions provided in the newick Utilities [25] package by modifying the source code provided in that package. We use *k* ∈ {3, 4, 5} and *c* ∈ {0, 1, 2, 3, 4, 5} in all of our experiments unless otherwise stated. In our experiments, we observed that the minimum overlap combination produced larger MFASTs than the in-order combination approach. Therefore, we limit our experimental results to the minimum overlap combination approach.

### Methods compared against

We have compared our method against Phylominer [20] and the MAST command implemented in PAUP* [26]. Among these, Phylominer also seeks MFASTs in a collection of trees. However, the time complexity of this method is exponential in the size of the input trees, and hence it becomes intractable for large trees. In our experiments, we observed that it does not scale beyond 50 taxa. PAUP* is primarily a program for phylogenetic inference, although it also can compute MASTs. MASTs have a strict 100% agreement criterion unlike the arbitrary frequency cutoff values *γ* in our method.

### Evaluation Criteria

We evaluate our algorithm based on the size of the MFAST found. Larger MFASTs are preferable. When possible, we report the size of the optimal solution as well.

### Test Environment

We ran our experiments on Linux servers equipped with dual AMD Opteron dual core processors running at 2.2 GHz and 3 GB of main memory to test the performance of our method.

### Datasets

We test the performance and verify the results of our method on synthetic datasets and real datasets. 

• Synthetic dataset We built synthetic datasets in which we embedded an MFAST as described below. We characterize each synthetic dataset using five parameters. The first two parameters denote the size and number of trees in T. MFAST frequency specifies the fraction of trees in T which contain an MFAST. MFAST size is the number of taxa in the embedded MFAST. The noise percentage is the percentage of taxa that is not a part of the embedded MFAST but is placed on the branches within the clade that contains the MFAST. We place all the other taxa on the branches outside this clade.Given an instantiation of these parameters, we first created a tree that has *n’* taxa. This tree serves as the MFAST. We then created *m* × *f* trees that contain this MFAST. We build each of these trees by inserting *n* − *n*^*′*^ taxa randomly in the MFAST. With probability ∊ we insert each taxa within the clade that contains MFAST. With probability 1 − ∊ we insert it outside that clade. We then created *m* − (*m* × *f*) trees that do not contain the current MFAST. We simply do this by inserting all the taxa one by one at a random location.

1. Tree size (*n*).

2. Number of trees (*m*).

3. MFAST frequency (*f*).

4. MFAST size (*n’*).

5. Noise percentage (∊).

• Real datasets. We use two empirical datasets to evaluate the performance of our heuristic. The data sets contain 200 bootstrap trees generated from phylogenetic analysis of the Gymnosperm [27] and Saxifragales (Burleigh, unpublished) plant clades. To make the bootstrap trees, we assembled super-matrices, matrices of concatenated gene alignments with partial taxon overlap, from gene sequence data available in GenBank. We performed a maximum likelihood bootstrap analysis on each super-matrix using RAxML v. 7.0.4 [28]. The Gymnosperm trees each contain 959 taxa, and the Saxifragales trees each contain 950 taxa.

### Effects of number of input trees

In our first experiment, we analyze how the number of input trees in T affects the performance of our algorithm. For this purpose, we created 30 synthetic datasets. The size of the embedded MFAST in all the datasets was 15. Among these 30 datasets, 10 contained 50 trees, 10 contained 100 trees and 10 contained 200 trees. We set the noise percentage to 20% in all the datasets. The frequency of the embedded MFAST was 0.8. We set the number of taxa in all the trees in these datasets to 100.

We ran our algorithm on each of these datasets to find the size of the MFAST for *γ* = 0.7. Table 2 lists the average MFAST size we found for each of the dataset sizes before post processing (i.e., at the end of phase two) and after post processing (i.e., at the end of phase three). The results demonstrate that our method can identify an MFAST that is almost as big as the embedded one even without post processing, regardless of the number of trees in the dataset. Post processing improves the MFAST size slightly. On the average, we always find an MFAST that is as large as or larger than the embedded one. An MFAST larger than 15 here implies that while randomly inserting the taxa that are not in the embedded MFAST, at least one of them was placed under the same clade at least a fraction *γ* of the time. More importantly, our method successfully located such taxa along with the rest of the MFAST.

**Table 2:** Evaluation of the effect of the number of trees in T

**Number of trees**	**MFAST size**	
	**Before post processing**	**After post processing**
50	14.5	16.0
100	15.3	15.8
200	14.4	15.4

The number of trees is set to 50, 100 and 200. For each number of trees we run our experiments on ten datasets. Each dataset contains trees with 100 taxa and an embedded MFAST of size 15. We report the average size of the MFAST obtained by our method across the ten datasets.

### Effects of tree size

Our second experiment considers the impact of the number of taxa in the input trees contained in T on the success of our method. To carry out this test, we built datasets with varying tree sizes (i.e., *n*). Particularly, we used *n* = 100, 250, 500 and 1000. For each value of *n*, we repeated the experiment 10 times by creating 10 datasets with the same properties. In all datasets, we set the number of trees to *m* = 100, the noise percentage at ∊ = 20%, the size of the embedded MFAST at 15% of *n*, and the MFAST frequency at 0.8.

Table 3 reports the average MFAST size found by our method for varying tree sizes. Second column shows the embedded MFAST size. Last two columns list the average size of the MFAST found by our method across the ten datasets. Before, going into detailed discussion of the results, it is crucial to observe that our method could run to completion for datasets that have as many as 1000 taxa. When we tried to run Phylominer, it did not return any results for datasets that have more than 100 taxa. The results also demonstrate that our method could successfully identify the embedded MFAST in all the datasets regardless of the size of the input trees. In some datasets, the reported MFAST was slightly larger than the embedded one. This indicates that while randomly inserting the taxa that are not part of the embedded MFAST, it is possible that a few taxa was consistently placed under the same same clade.

**Table 3:** Evaluation of the effect of the size of the trees in T

**Number of**	**MFAST size**		
**taxa**	**Embedded**	**Reported**	
		**Before post**	**After post**
		**processing**	**processing**
100	15	15.3	15.8
250	38	32.3	38.8
500	75	43.7	76.0
1000	150	69.8	151.0

The tree size is set to 100, 250, 500 and 1000. For each tree size we run our experiments on ten datasets. Each dataset contains 100 trees with an embedded MFAST of size 15% of the input tree size. Second column shows the embedded MFAST size. Last two columns list the average size of the MFAST found by our method across the ten datasets.

The results also suggest that our method identifies a significant percentage of the taxa in the embedded MFAST after the second phase (i.e., before post-processing) when the tree size is small. As the tree size grows, it starts missing some taxa at this phase. It however recovers the missing taxa during the post-processing phase even for the largest tree size. This indicates that at the end of phase two our method could identify a backbone of the actual MFAST. The unidentified taxa at this phase are scattered throughout the clades in the input trees. Thus, there is no clade of size *k* + *c* that contains them with *c* contractions for small *k* or *c*. As evident from Table 3, this however does not prevent our method from recovering them. This is because the backbone reported at the end of phase two is large enough, and thus specific enough, to recover the missing taxa one by one in the last phase. This is a significant observation as it demonstrates that our method works well even with small values of *k* and *c*.

### Effects of noise percentage

Recall that the noise percentage ∊denotes the percentage of taxa that is added inside the clade that contains the MFAST. As ∊increases, the pairs of taxa in the MFAST get farther away from each other in the tree that contains it. As a result, fewer taxa from MFAST will be contained in small clades of size *k* + *c*. This raises the question whether our method works well as ∊increase and thus the MFAST taxa gets scattered around in the trees that contain it.

In this experiment, we answer the question above and analyze the effect of the noise percentage on the success of our method. We create synthetic datasets with various ∊ values. Particularly, we use ∊ = 20, 40 and 60%. We set the size of the embedded MFAST to *n*^*′*^ = 15, the tree size to *n* = 100, number of trees to *m* = 100 and the MFAST frequency to *f* = 0.8. We repeat our experiment for each parameter 10 times by recreating the dataset randomly using the same parameters. We set the frequency cutoff to *γ* = 0.7. We report the average MFAST size found by our method in Table 4.

**Table 4:** Evaluation of the effect of the noise in the trees in T

**Noise (%)**	**MFAST size**	
	**Before post processing**	**After post processing**
20	15.3	15.8
40	13.6	15.0
60	12.7	15.0

The size of the embedded MFAST in all the experiments is 15. We list the average size of the MFAST found by our method before and after the post processing phase.

The results suggest that our method can identify the embedded MFAST successfully even when the noise percentage is very high. We observe that the size of the MFAST found by our method before post processing decreases slowly with increasing amount of noise. This is not surprising as the taxa contained in the embedded MFAST gets more spread out (and thus farther away from each other) in the trees in T with increasing noise. As a result, if there are taxa that are not part of any seed with the provided values of *k* and *c*, they will never be included in the computed MFAST at the end of phase two. We however observe that (i) only a small number of such taxa exists. For instance, even for the largest noise percentage (∊ = 60%), only 2.3 taxa (i.e., 15 - 12.7) are missing on the average. (ii) The missing taxa are recovered during phase three. This is because the computed MFAST at the end of phase two is very large, and thus it is specific to the embedded MFAST.

### Impact of seed creation

So far, in our experiments we consistently observed two major points for all the parameter settings (see Sections “Effects of number of input trees” to “Effects of noise percentage”): (i) Our method always finds a large subtree of the embedded MFAST after phase two. (ii) Our method always recovers the entire embedded MFAST after phase three. The second observation can be explained from the first one that the outcome of phase two is large enough to build the entire MFAST precisely. The first observation however indicates that the set of seeds generated in phase one contain a significant percentage of the taxa in the embedded MFAST. In this section, we take a closer look into this phenomenon and explain why this is the case even for small values of seed size *k* and contraction amount *c*, and large noise percentage ∊. To do that, we will compute the probability that a subset of the taxa of the embedded MFAST appears in at least one seed generated in phase one. In our computation, we will assume that the taxa can appear at any location of a given tree with the same probability. We discuss the implication of this assumption later in this section.

The number of rooted bifurcating trees for a given set of *n* taxa is

$$\;R\left(n\right)=\frac{(2n-3)!}{2^{n-2}(n-2)!}.$$

Consider a clade with *k* + *c* taxa. The number of trees with *n* taxa that contains this clade is *R*(*n* − (*k* + *c*) + 1) as the topology of the *k* + *c* sized clade is fixed. For a given a subtree with *k* taxa, let us denote the number of clade topologies of size *k* + *c* that contains that subtree with NU(*k*, *c*). We can compute this function recursively as NU(*k*, 0) = 1 and for *c* > 0,

NU(k,c)=NU(k,c−1)×2×(k+c−2).

Let us denote one of these clades by U(*k*, *c*). Also, let us denote the probability that the clade U(*k*, *c*) exists in a random tree topology that contains *n* taxa with *P*(*n*, *k*, *c*). Intuitively, *P*(*n*,*k*,*c*) is the probability that our method will extract a specific *k* taxa subtree from one *n* taxa tree after only *c* contractions. We can compute this probability as the ratio of the number of tree topologies that satisfy this constraint to that of all possible tree topologies. We formulate this as

$$\;P\left(n,k,c\right)=\frac{\mathrm{NU}(k,c)\times R(n-(k+c)+1)}{R(n)}.$$

Recall that it suffices for our algorithm to have a *k* taxa subtree of the MFAST in at least one tree in the given set of *m* trees. The probability that the clade U(*k*, *c*) exists in at least one of the *m* random tree topologies each containing *n* taxa is

$$\;P(n,k,c,m)=1-{\left(1-P(n,k,c)\right)}^{m}.$$

Assume that the MFAST size in the given set of trees T is *h*. Let us denote the number of *k* taxa subtrees of the MFAST as NS(*h*, *k*). The probability that at least one of these subtrees will be found in at least one of the input trees is then

$$\;P(n,k,c,m,h)=1-{\left(1-P(n,k,c,m)\right)}^{\mathrm{NS}(h,k)}.$$

A lower bound to NS(*h*, *k*) is *h* − *k* + 1 which can be obtained by picking a contiguous block of *k* taxa from the canonical newick representation of the MFAST by considering all possible *h* − *k* + 1 starting point locations. Notice that the larger the value of *P*(*n*, *k*,*c*, *m*, *h*), the higher the chances that our algorithm will construct some part of the MFAST. Similarly, the larger the value of NS(*h*, *k*), the higher the chances that our algorithm will construct some part of the MFAST.

**Figure 6:**
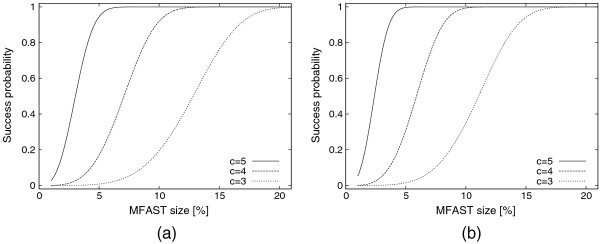
**The probability of finding at least one seed which contains a part of an MFAST**. The number of contractions *c* is set to 3, 4 and 5 and the corresponding seed size *k* is 5, 4 or 3. The x-axis shows the MFAST size in terms of the percentage of the number of taxa in the trees in T. In (**a**), we set the total number of trees *m* = 500. In (**b**) we set *m* = 1000.

Figure 6 plots the success probability (i.e., *P*(*n, k, c, m, h*)) of our method for varying parameter values. As the MFAST size increases, the success probability rapidly increases. This is because the number of alternative subtrees of the MFAST increases with increasing MFAST size. Thus, the chance of observing at least one increases as well. We observe that when the size of MFAST is around 20% of the tree size, for all the parameters reported our success probability becomes almost 1. As the number of contractions increases, the probability of success increases. This is because large number of contractions increases the possibility of eliminating false positive taxa from clades. In other words, it helps gluing the taxa that are normally scattered in the input trees back together by removing the remaining taxa among them. When *c* = 5, our success probability becomes almost one even for MFASTs that are as small as a 4-6% of the tree size. As the number of trees increases, the success probability increases as well. This is because we have more alternative topologies with increasing number of trees. Thus, there are more chances to have a small clade that contains a part of the MFAST. Finally, it is worth noting that these results are computed based on the assumption that the trees in T are uniformly distributed among all possible topologies. In practice, we expect that these trees are constructed with the same or similar objectives (such as maximum parsimony or maximum likelihood). As a result, they will likely have a higher chance to contain large MFASTs. The results we expect in practice will thus be similar or even better than the theoretical results in Figure 6.

Overall, we conclude from this experiment that even small values of *k* and *c* suffices to capture a part of the MFAST in phase two. Therefore, although our algorithm’s complexity increases exponentially with *k* and *c*, we do not need to use large values for *k* and *c*. *This enables our algorithm to scale to very large datasets with thousands of taxa and trees. These results explain the theory behind the practical results we observed in Sections “Effects of number of input trees” to “Effects of noise percentage”.*

### Evaluation of state of the art methods

So far, we have shown that our method could successfully find the MFASTs contained in sets of trees T for up to 1000 taxa and 200 trees (i.e., *n* = 1000 and *m* = 200). An obvious question is how well do existing methods perform on the same datasets. Here, we answer this question for two existing programs, namely PAUP* (version 4.0b10) and Phylominer.

When we fix the number of trees and the number of taxa to 100, PAUP* was able to find the MAST for for all datasets. As we grow the number of taxa to 250 or larger while keeping the number of trees as 100, PAUP* runs our of memory and fails to return any results. After reducing the number of trees to 50, PAUP* still runs out of memory and cannot report any results for more than 100 taxa.

The scalability problem of Phylominer is even more severe. Phylominer is able to compute the MFASTs on datasets with up to 20 taxa. However, as we increase the number of taxa further, its performance deteriorates quickly. When we set the number of taxa to 100, even with as few as 100 trees, Phylominer takes more than a week to report a result. Moreover, in our experiments, the maximum size of the subtrees it found on average contained fewer than 7 taxa, even though the size of the true MAST was 10.

Another interesting question about existing methods would be whether the majority consensus rule can be used to find MFASTs. To evaluate this, we used the same three synthetic datasets used in Section “Effects of noise percentage”. Recall each of these three datasets contains an MFAST of size 15 which is embedded in 80% of the trees. The datasets are created with 20%, 40% and 60% noise indicating different levels of difficulty in recovering the embedded MFAST. We computed 70% majority consensus tree. Notice that if majority consensus rule can identify an MFAST, that would correspond to a bifurcating subtree topology in the consensus tree. In other words a subtree is bifurcating in this experiment only if 70% or more of the input trees agree on the topology of that subtree. The resulting tree, however, was multifurcating for all the three datasets. This means that majority consensus rule could not recover even a smaller portion of the embedded tree while our method was able to locate the entire MFAST successfully (see Table 4).

These results demonstrate that both PAUP* and Phylominer are not well suited to finding agreement subtrees in larger datasets, our method scales better in terms of both the number of taxa and the number of trees. When PAUP* runs to completion, we observed that it reports the true results. Recall from previous experiments that our method always found the true results on the same datasets as well as larger datasets. *This suggests that our method has the potential to have an impact in large scale phylogenetic analysis when existing methods fail. *

### Empirical dataset experiments

To examine the performance of the MFAST method on real data, we performed experiments using 200 maximum likelihood bootstrap trees from a phylogenetic analysis of gymnosperms (959 taxa) and Saxifragales (950 taxa). Specifically, we evaluated how the performance of the MFAST algorithm was affected by the number of input trees and the size of the input trees.

#### Effects of number of input trees

We first examined the effect of input tree number on the size of MFAST. For both the gymnosperm and Saxifragales trees, we generated 10 sets of 50 and 100 trees by randomly sampling from the original 200 trees without replacement. We compared the average size of the MFAST in the 50 and 100 tree data sets with the size of the MFAST in the original 200 tree data set. First, in all analysis, the post-processing step greatly increases the size of the MFAST, sometimes more than doubling it (Table 5). This increase is similar to the one observed in the 1000 taxon simulated data sets (Table 2), emphasizing again the importance of the post-processing step with large tree data sets. Although the sizes of the MFASTs were similar, they decreased slightly with the addition of more trees (Table 5). This may simply be a matter of observing more conflict with more trees.

**Table 5:** The size of the MFAST found by our method on the Gymnosperms and Saxifragales datasets before and after post processing (phase three)

**Number of**	**MFAST size**					
**trees**	**Gymnosperms**			**Saxifragales**		
	**Before**	**After**	**Only**	**Before**	**After**	**Only**
50	78.5	129.8	99.5	64.7	122.0	84.1
100	68.4	119.2	83.1	55.4	112.8	74.7
200	76.0	118.0	84.0	40.0	105.0	75.0

The size of the MFAST found by running only the post processing step is also shown. We run our method on the entire dataset that contains 200 trees as well as randomly selected subsets of 50 and 100 trees. We repeated the 50 and 100 tree experiments 10 times by randomly selecting the trees from the entire dataset and reported the average value.

The large gap between the MFAST sizes before and after the post processing suggests that phase three is the main reason behind the success of our method, and thus, the costly seed combination phase (i.e., phase two) may be unnecessary. To answer whether this conjecture is correct, we ran a variant of our method by disabling the second phase; we only ran the post processing phase starting from each seed as the initial MFAST one by one. We reported the largest MFAST found that way as the output of this variant in Table 5. The results demonstrate that although phase three can grow a large FAST, *phase two is essential* to find the largest frequent agreement subtree. In other words, post processing finds the true MFAST only if a large portion of it is already found (which is the role served by phase two). In conclusion, phase three of our method cannot replace phase two, yet both phases are essential for the success of our method.

#### Effects of size of input tree

Next, we examined the effect of number of leaves in the input trees on the size of MFASTs. For both the gymnosperm and Saxifragales trees, we generated 10 sets of 200 input trees with 100, 250, and 500 taxa. To make each set, we randomly selected 100, 250, or 500 taxa, and we deleted all other taxa from the original sets of 200 trees. Thus, these sets of trees with 100, 250, or 500 taxa are subtrees of the original data sets. The size of the average MFAST increases with more taxa in the original trees (Table 6). However, interestingly, the average size of the MFASTs for the gymnosperm data set with 500 trees is larger than the MFAST found from the original gymnosperm trees with all the taxa (Table 6). Since the MFAST from the 500 taxon data sets should all be found within the full data set, this indicates that on the larger trees, our method may not always find the true (i.e., largest) MFAST. The full data sets may require a larger number of contractions to find the true MFASTs.

**Table 6:** The size of the MFAST found by our method on the Gymnosperms and Saxifragales datasets before and after post processing (phase three) for different number of taxa

**Number of**	**MFAST size**					
**leaves**	**Gymnosperms**			**Saxifragales**		
	**Before**	**After**	**Only**	**Before**	**After**	**Only**
100	41.2	56.1	43.5	43.5	50.7	38.5
250	67.2	88.5	63.0	62.3	76.2	54.6
500	91.6	123.0	74.9	52.0	86.7	62.9
All	76.0	118.0	84.0	40.0	105.0	75.0

The size of the MFAST found by running only the post processing step is also shown. We run our method on the entire dataset that contains all the taxa (last row) as well as randomly selected taxa subsets of size 100, 250 and 500. We repeated the 50, 100 and 250 taxa experiments 10 times by randomly selecting the taxa from the entire dataset and reported the average value.

Similar to the experiments in Section “Effects of number of input trees”, we investigated the gap between the MFAST sizes before and after the post processing step. We ran a variant of our method by disabling the second phase; we only ran the post processing phase starting from each seed as the initial MFAST one by one. We reported the largest MFAST found that way as the output of this variant in Table 6. The results are in parallel with those in Table 5. Phase three can grow a large frequent agreement subtree, but not quite as big as that when both phase two and three are executed.

#### Effects of sample size

In our final experiment, we evaluated the effect of the *maximum time cutoff *, we described in Section “In-order combination” on the accuracy of our method. Recall that, this cutoff limits the number of initial seeds tried in our algorithm by randomly sampling a small percentage of the seeds. It only uses the sampled seeds as possible initial seeds. However, it uses the entire set of seeds while growing the MFAST determined by the initial seed. As each initial seed roughly takes the same amount of time to grow into an MFAST, using *x*% of the seeds as the sample set reduces the total running time our method to roughly *x*% of that of our original implementation.

We carried out this experiment as follows. For both the gymnosperm and Saxifragales trees, we ran 10 sets of experiments for each sampling percentage of 2, 5, 10, 25, 50 and 100%. Thus, totally we ran 60 (6 × 10) experiments. Table 7 presents the average MFAST sizes for varying sample sizes. The results demonstrate that even for very small sampling percentages, our method finds MFAST that is almost as big as the MFAST found by using the entire dataset (i.e., 100% sampling percentage). This is very promising as it demonstrates that the running time cost of our method can easily be cut to a small fraction by sampling the starting seeds. The rationale behind this is that the MFAST contains many seeds. Starting from any of these seeds, our algorithm has the potential to lead to that MFAST. The probability that at least one of these seeds appear in the sample set is large particularly for large MFASTs.

**Table 7:** The size of the MFAST found by our method on the Gymnosperms and Saxifragales datasets for different random subsamples of the total number of taxa

**Sampling**	**MFAST size**	
**percentage**	**Gymnosperms**	**Saxifragales**
2	85.9	74.3
5	87.5	75.6
10	88.4	75.2
25	87.5	75.5
50	88.5	76.2
100	88.5	76.2

We run our method by randomly picking 2%, 5%, 10%, 25%, 50%, 100% of the seeds found in phase one for combination in phase two.

## Conclusion

In this paper, we present a heuristic for finding the maximum agreement subtrees. The heuristic uses a multi-step approach which first identifies small candidate subtrees (called seeds), from the set of input trees, combines the seeds to build larger candidate MFASTs, and then performs a post-processing step to increase the size of the candidate MFASTs. We demonstrate that this heuristic can easily handle data sets with 1000 taxa, greatly extending the estimation of MFASTs beyond current methods. Although this heuristic is not guaranteed to find all MFASTs, it performs well using both simulated and empirical data sets. Its performance is relatively robust to the number of input trees and the size of the input trees, although with the larger data sets, the post processing step becomes more important. Overall this method provides a simple and fast way to identify strongly supported subtrees within large phylogenetic hypotheses.

Although the method we developed is described and implemented for the rooted and bifurcating trees, it can be trivially extended to multifurcating as well as unrooted trees. The central technical difference in the case of unrooted trees would be the definition of clade (see Definition 1) as the definition requires a root. A clade in an unrooted tree encompasses two sets of nodes; (i) a given set of taxa *X*, (ii) the set of all internal nodes that are on a path between two taxa in *X* on the phylogenetic tree. We expect that this will increase the number of seeds substantially and thus make the problem more computationally intensive. The amount of increase will depend on the tree topology. The theoretical worst case happens when all the taxa are connected to a single internal node (i.e., star topology). In that case any subset of taxa can lead to a potential seed as long as the subset size is equal to the seed size allowed. One possible way to overcome this problem would be to exploit randomization or graph coloring strategies and avoid enumerating majority of the possible seeds.

## Abbreviations

MAST: Maximum agreement subtree; FAST: Frequent agreement subtree; MFAST: Maximum frequent agreement subtree.

## Competing interests

The authors declare that they have no competing interests.

## Authors’ contributions

AR participated in algorithm development, implementation, experimental evaluation and writing of the paper. TK participated in algorithm development, experiment design and writing of the paper. GB participated in experiment design, dataset collection and writing of the paper. All authors read and approved the final manuscript.
